# Festina Lente

**Published:** 1883-11

**Authors:** Wm. Jefferson Guernsey


### FESTINA LENTE.

Wm. Jefferson Guernsey, M. D.

The following rules are gathered from observations by men who *ought* to know, and whose success in practice proves that they *do* know, "whereof they speak."

Sympathy for the sufferings of those intrusted to our care, anxious entreaties of friends and relatives and the gibes of skeptical neighbors, all tempt the worried doctor to "hurry up the cure." As a check on this pressure to such ruinous haste, let the reader cut these *rules* out and paste them in his *Materia Medica* and there review them from time to time.

'Tis here that the adage quoted above is proven a truth in verity.

The writer has repeatedly verified every one of the rules, and only regret that he has so many times, to his sorrow, neglected to be led by them.

RULES.

1. *Never* prescribe until you are *sure* of your choice of a remedy. (Study the case at the bedside, or give S. L. and wait till you reach your office, but do *not* give a *temporary* medicine. The *first* prescription may " make or break.")

2. In the first prescription, or subsequently on changing to another remedy, give but *one* dose and wait. (One dose will often cure a case. If it does not more can easily be supplied. Some cases are very susceptible and easily aggravated, which may confuse you and must delay the cure.)

3. If found necessary to *repeat* a medicine already prescribed, give several doses of the same potency in water, or of a different potency dry.

4. After repeating a medicine, allow an interval of *rest* without medicine.

5. Let the patient have plenty of Sac. Lac. (How can we expect the laity to have faith in the " one dose " system when so many of the profession ridicule it?)

6. Give no medicine so long as the patient continues to improve.

7. Do not be tempted to deviate from Rule 6 because *new* symptoms arise if the patient otherwise is *really* better.

8. So long as the patient grows *no worse, even if not better*, in a disease that would probably increase in severity without treatment, it is *favorable* and should go without medicine. (Dr. C. Lippe recently related to the writer a severe case of dysentery for which, at the first prescription, he gave one dose of a remedy well indicated. The patient, who had previously been growing rapidly worse, was found the next day at a *statu quo*. No medicine was given. The next day discovered precisely the same state. No medicine. On the following day the case was almost well. A great many remedies require a rest, and as the *aggravation* had ceased, the Doctor rightly concluded he was " making his point.")

9. If a relapse into the same symptoms follow an amelioration from the single dose, that remedy must be repeated. (See Rule 3.)

10. If *new* and important symptoms appear, be *sure beyond a doubt* whether they do not belong to the remedy just given. If they do, *wait*. (These new symptoms may be an aggravation of the remedy. If you cannot remember them as belonging under that drug, look it up rather than spoil the case by a change.)

11. If there is no improvement and there has not been any, and the case is one that would probably remain so without treatment, review the symptoms to see whether the remedy last given is still indicated before changing to another. If you are satisfied with the *first* choice, *repeat* it as suggested in Rule 3.

12. If the disease is a "periodic" one it is *favorable* if the next attack following the administration of the first dose is in the *slightest degree* lighter, shorter, or later; or if it is *markedly* the *reverse*, viz.: *very much* heavier, longer, or earlier. In either case, *wait*.

